# Global proteomics dataset of miR-126 overexpression in acute myeloid leukemia

**DOI:** 10.1016/j.dib.2016.07.035

**Published:** 2016-08-24

**Authors:** Erwin M. Schoof, Eric R. Lechman, John E. Dick

**Affiliations:** a Princess Margaret Cancer Centre, University Health Network, Canada; b Department of Molecular Genetics, University of Toronto, Toronto, Canada ON M5G 1L7

**Keywords:** Acute myeloid leukemia, Proteomics, miRNA, FACS sorting

## Abstract

A deep proteomics analysis was conducted on a primary acute myeloid leukemia culture system to identify potential protein targets regulated by miR-126. Leukemia cells were transduced either with an empty control lentivirus or one containing the sequence for miR-126, and resulting cells were analyzed using ultra-high performance liquid chromatography (UHPLC) coupled with high resolution mass spectrometry. The mass spectrometry data have been deposited to the ProteomeXchange Consortium via the PRIDE partner repository with the dataset identifier PRIDE: PXD001994. The proteomics data and statistical analysis described in this article is associated with a research article, “miR-126 regulates distinct self-renewal outcomes in normal and malignant hematopoietic stem cells” (Lechman et al., 2016) [1], and serves as a resource for researchers working in the field of microRNAs and their regulation of protein levels.

**Specifications Table**

**Table:** 

Subject area	*Biology*
More specific subject area	*Acute Myeloid Leukemia, microRNA*
Type of data	*Figures, Perseus workflow, R script*
How data was acquired	*LC MS/MS on an Orbitrap Fusion Mass Spectrometer (Thermo Fisher Scientific)*
Data format	*RAW, filtered and analyzed*
Experimental factors	*Samples were subjected to SCX fractionation prior to analysis*
Experimental features	*miR-126 was overexpressed in a primary AML culture system through viral transduction, and samples were analyzed and compared between miR-126 and empty control viral vectors.*
Data source location	*University Health Network, Toronto, Canada*
Data accessibility	*Data is within this article and the mass spectrometry data have been deposited to the ProteomeXchange Consortium via the PRIDE partner repository with the dataset identifier PRIDE:*PXD001994 (http://proteomecentral.proteomexchange.org/cgi/GetDataset?ID=PXD001994)

**Value of the data**

• First global proteomics dataset of miR-126 overexpression in the context of primary human leukemic cells.
• Enforced expression data sheds first light on miR-126 driven protein regulation for use by leukemia researchers.
• Targets highlighted by proteomics data provide the community with candidates for proteins under (direct) control of miR-126.

## 1 Data

The dataset described in this article embodies the first global proteomics dataset investigating the biological impact of miR-126 enforced expression in human AML cells. The data files shared here provide the computational workflow that was applied to filter the data in Perseus [2], and to determine significantly regulated proteins using Limma [6]. Furthermore, the experimental workflow and an overview of the technical and biological reproducibility of the analyses are presented.

## 2 Experimental design, materials and methods

**Fig. 1:**
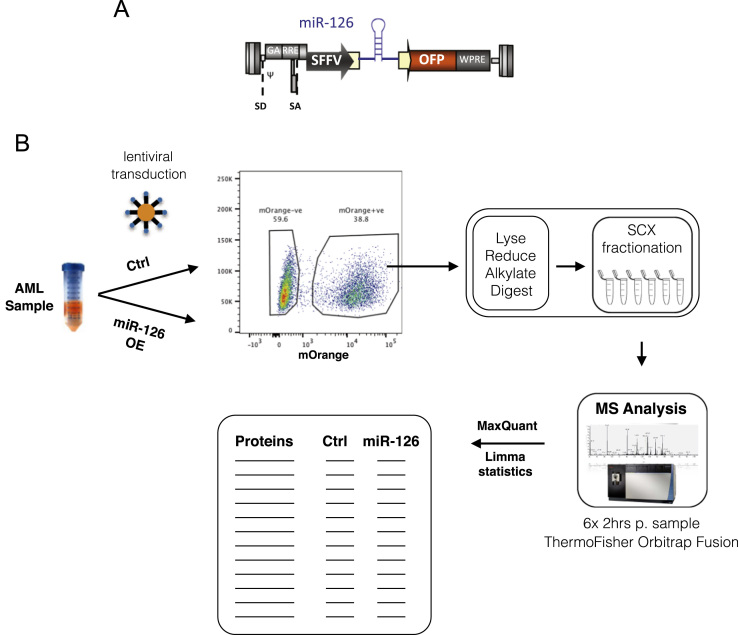
(A) Schematic representation of the lentiviral construct for enforced expression of miR-126. The human miR-126 coding sequence is driven off of the SFFV promoter. (B) Experimental workflow for generation of proteomics data from cells transduced with miR-126 and CTRL virus. Two weeks after viral transduction, mOrange positive cells are sorted, and after cell lysis, proteins are reduced, alkylated and digested, and subsequently subjected to SCX fractionation for deep proteome coverage. Resulting peptide fractions are analyzed on an Orbitrap Fusion and the raw data is interpreted using MaxQuant. Resulting protein expression levels are tested for significance in Limma, resulting in a final quantitative table of comparative protein expression levels between miR-126OE and CTRL.

To assess the protein-level regulation of direct targets of miR-126, we conducted a proteomics analysis to compare AML cells transduced with either a miR-126 overexpression (126OE) or control (CTRL) vector (Fig. 1A and B). A primary AML culture system, 8227 (described in [1]), was subjected to viral transduction and cells were subsequently analyzed for their global protein expression levels using mass spectrometry. Deep proteome coverage was obtained through the use of SCX fractionation, and protein quantitation was conducted using a label-free quantitation (LFQ) approach [3].

Two weeks postviral transduction, three biologically independent sets of 8227 cells transduced with either 126OE and CTRL vectors (also containing the mOrange gene to enable detection of transduced cells) were flow sorted for mOrange^+^ cells, counted and subjected to sample preparation as described in [1]. Briefly, cells were lysed, boiled at 95 °C and sonicated, to subsequently be digested in a 2-step digestion protocol with Lysyl Endopeptidase C (MS grade, Wako) and Trypsin (MS grade, Promega). Resulting peptide samples were simultaneously desalted and fractionated using Strong Cation Exchange StageTips (2251, Empore 3M) packed in-house [4]. Five fractions were eluted using 50, 75, 125, 200 and 300 mM ammonium acetate in 20% Acetonitrile, 0.5% formic acid respectively, and the final fraction was eluted using 5% ammonium hydroxide, 80% Acetonitrile. After concentrating the samples in an Eppendorf Speedvac, the eluted fractions were re-constituted in 1% TFA, 2% Acetonitrile for Mass Spectrometry (MS) analysis.

### 2.1 Mass spectrometry acquisition

**Fig. 2:**
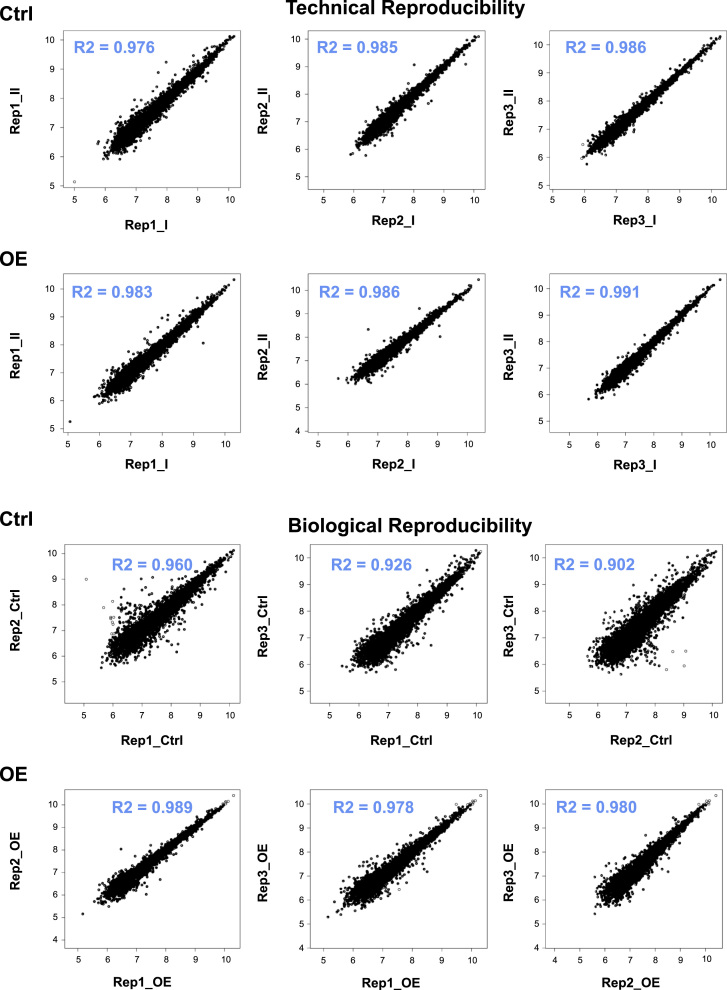
Overview of technical and biological reproducibility of the mass spectrometry analyses.

Each SCX fraction was analyzed on an Orbitrap Fusion (Thermo Fisher Scientific), connected to a Thermo EasyLC 1000 UHPLC system in a single-column setup, and peptides were eluted over a 140 min gradient on a 50 cm C18 reverse-phase analytical column (Thermo Fisher EasySpray ES803). Detailed MS settings are described in [1], and mass spectrometry performance was monitored for consistency throughout the analysis of standard QC samples generated from complex HEK293T lysates. Each sample was run in technical duplicate, and the reproducibility of the analyses is depicted in Fig. 2. All raw files were deposited to the ProteomeXchange Consortium via the PRIDE partner repository with the dataset identifier PRIDE: PXD001994 [5].

### 2.2 Label-free quantitative proteomics analysis

MaxQuant version 1.5.2.8 [3] was used to analyze the resulting .raw files and generate the label-free quantitation (LFQ) values. A minimum of 3 unique peptides per protein was required, and Oxidation (M), Acetyl (protein N-term), Gln->pyro-Glu and Glu->pyro-Glu were set as variable modifications. False discovery rate was kept constant at 1%, and “match between runs” was enabled.

The resulting table, containing all identified proteins and LFQ values was processed in Perseus (version 1.5.0.9, workflow attached in Supplementary materials) [2]. After removing contaminants and reverse hits, 8848 proteins remained, of which 4837 proteins were quantified in all samples. Protein ratios for each biological replicate were calculated, and this final table was processed in Limma (R Statistical Framework [6]) to determine those proteins that are significantly regulated according to the moderated *t*-test. Limma input, the R script and results are attached in this manuscript, and the final results used for downstream analysis can be found as Table S4 in [1].
